# Massive Submandibular Sialolith: Complete Radiographic Registration and Biochemical Analysis through X-Ray Diffraction

**DOI:** 10.1155/2014/659270

**Published:** 2014-09-02

**Authors:** Ademir Franco, Mayara Jessica de Carvalho Mattos, Francine Ferrari, José Manoel dos Reis Neto, Luiz Carlos Carta Gambus, Paulo Henrique Couto Souza, Soraya de Azambuja Berti-Couto

**Affiliations:** ^1^Departamento de Estomatologia, Escola de Saúde e Biociências, Pontifícia Universidade Católica do Paraná, Imaculada Conceição 1155, Prado Velho, 80215-901 Curitiba, PR, Brazil; ^2^Laboratório de Análise de Minerais e Rochas, Departamento de Geologia, Universidade Federal do Paraná, Centro Politécnico 19062, Jardim das Américas, 81615-980 Curitiba, PR, Brazil

## Abstract

Sialolithiasis is a pathologic condition that affects 60 million people per year, which is caused by the presence of calcified structures, named sialoliths, inside the salivary glands and their salivary ducts. Despite the large incidence of sialolithiasis, its etiology is still unknown. In the present case report, a 47-year-old female patient, presenting with local pain and hampered mouth opening, underwent a surgical approach for the removal of a 20 mm sialolith, which was further analyzed through X-ray diffraction. In parallel, a radiographic registration of 8 years, covering all the period for sialolith formation, is presented along the case report.

## 1. Introduction

The salivary calculi are calcified structures often referred to as sialoliths, which consist of minerals such as calcium phosphate and hydroxyapatite, as well as other substances such as magnesium, potassium, and ammonia [1]. Sialoliths often lead to sialolithiasis, which is a common disease, affecting around 60 million people per year [1]. Mostly, sialolithiasis is clinically characterized by local pain and edema, reduced salivary flow, hampered mouth opening, spontaneous bleeding, and purulent discharge [2]. Radiographically, rounded or cylindrical radiopaque structures are observed near to the salivary glands, or their ducts, especially in panoramic and occlusal radiographs [3].

Small sialoliths can be spontaneously expelled through the stimulation of the salivary flow by performing local massage or using mechanical or chemical sialogogues, such as bubble gums and citric acid, respectively [4]. On the other hand, multiple or massive sialoliths often require major approaches, such as lithotripsy, sialadenectomy, sialotomy, and sialodochoplasty [3].

The present study aims to report the case of a 47-year-old female patient, who was diagnosed with sialolithiasis based on clinical and radiographic signs. Specifically, the present case is illustrated by a minimally invasive surgical procedure, a radiographic registration of 8 years covering the entire period of a massive sialolith formation, and a biochemical analysis through X-ray diffraction.

## 2. Case Report

**Figure 1:**
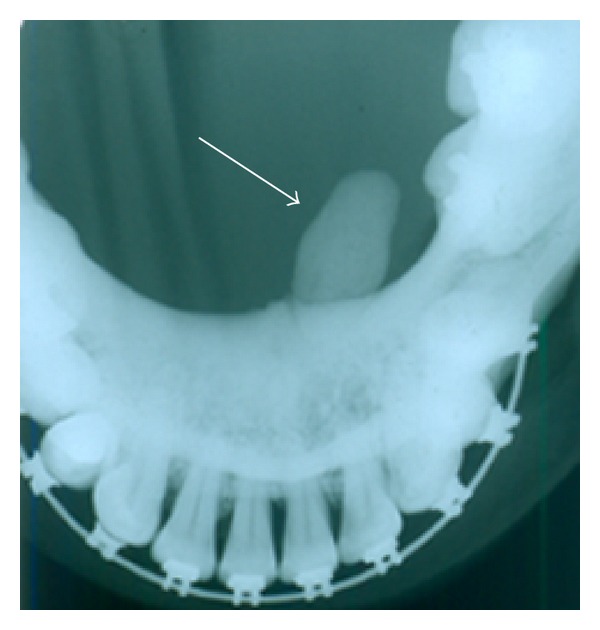
Occlusal radiograph of the massive sialolith (arrow).

In August 2012, a 47-year-old, white Caucasian, female patient was referred, by an orthodontist, to the Stomatology Department of the Pontifícia Universidade Católica do Paraná, Brazil, presenting with extreme signs of local pain in the submandibular region and speaking limitations due to hampered mouth opening. Clinically, left submandibular lymphadenopathy was detected, as well as edema in the left side of the floor of the mouth, with a purulent discharge. In addition, a yellowish structure of hard consistency was observed near to the sublingual caruncle. An occlusal radiograph revealed a cylindrical radiopaque sialolith-compatible image, measuring approximately 2 × 1 cm, in the lower left canine region, confirming the diagnosis of submandibular sialolithiasis (Figure 1).

**Figure 2:**
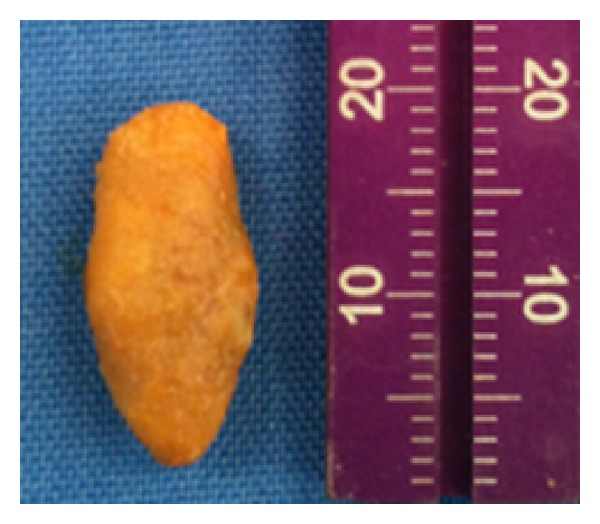
Clinical view of the sialolith after surgical removal.

During the anamnesis, the patient did not report systemic diseases and reported being allergic to Penicillin. Further, the surgical excision was executed in the same day due to the exacerbated symptoms and the favorable position, in which the sialolith took place after a submandibular massage. Under local anesthesia, a 5 mm incision was performed on the mucosa, over the sialolith. In order to move the sialolith close to the incision, the submandibular massage in the posteroanterior direction was repeated. Using forceps, a 20 mm sialolith (Figure 2) was removed from the floor of the mouth. After the sialolith removal, it was possible to see the reinstatement of salivary flow and also the reduction of the purulent discharge. The patient was medicated with Azithromycin 500 mg, Ibuprofen 600 mg, and Paracetamol 750 mg.

**Figure 3:**
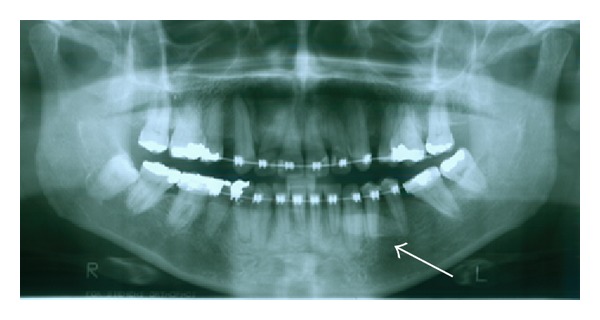
The last panoramic radiograph previously obtained for orthodontic purposes indicating the massive sialolith (arrow).

**Figure 4:**
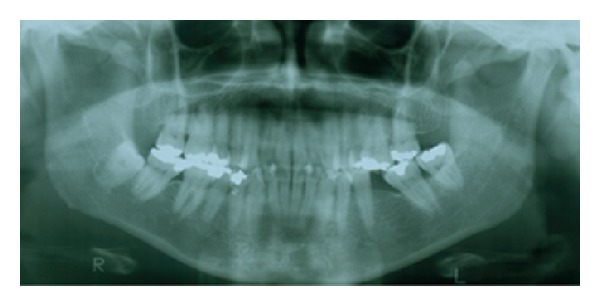
An additional panoramic radiograph retrieved from the patient's file revealing no sign of sialolith formation.

In the following week, the patient returned without edema and local inflammation. The submandibular gland function was tested performing local massage, indicating normal salivary flow. Surprisingly, the patient provided a previous panoramic radiograph, dating from March 2011 (Figure 3), representing the last radiographic exam for conclusion of orthodontic treatment. Despite slight morphological alteration, the orthodontic radiograph allowed for the detection of the sialolith. Based on the lack of early detecting the sialolith on the orthodontic radiograph, a deeper investigation in the patient's dental files was carried out revealing an additional panoramic radiograph, dating from June 2004, in which no sign of sialolith was detected (Figure 4). Clinical and radiographic followup were performed 6 months after the surgery revealing no alterations.

## 3. Biochemical Analysis

**Figure 5:**
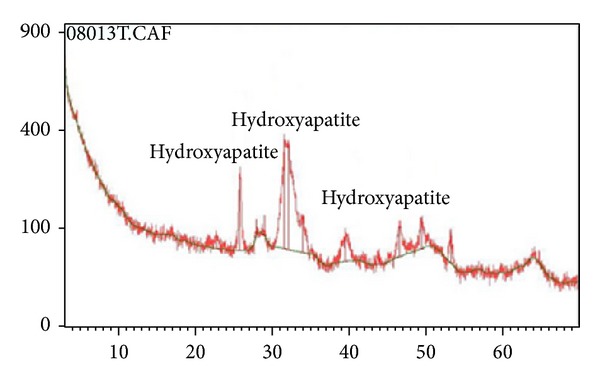
Outcome from the analysis through X-ray diffraction indicating hydroxyapatite as mineral structure of the massive sialolith.

The sialolith was referred to the Laboratory for Analysis of Minerals and Rocks, Geology Department, Universidade Federal do Paraná, Brazil (LAMIR-UFPR), for biochemical analysis through X-ray diffraction. The stone weighted 0,593 g. An Empyrean Diffractometer (PANalytical, Almelo, the Netherlands) processed the powdered sialolith. The measured scattered pattern produced out of the interaction between the X-ray beam and the sialolith surface indicated hydroxyapatite Ca5(PO4)(CO3)3(OH) as the only mineral present (Figure 5).

## 4. Discussion

The medical literature reports the submandibular salivary glands as the most commonly related pair of glands in cases of sialolithiasis (around 80% of prevalence) [5–7], specially involving massive sialoliths [8]. It is explained by the submandibular salivary duct morphology, a tortuous structure which links the salivary gland to the oral cavity. The most narrowed path of the referred duct is named “comma area,” which is located near to the duct's outfall and facilitates the deposition of minerals, such as calcium, creating a proper nidus for sialolith formation [8, 9]. In addition, the submandibular salivary gland presents an alkaline environment with high concentration of phosphate, which contributes for the formation of hydroxyapatite. In previous studies [10, 11], hydroxyapatite was detected through X-ray diffraction and classified as the main mineral structure in 24 out of 27 sialoliths. The remaining sialoliths were composed of organic structures such as epithelial cells and bacteria. Accordingly, the present study indicates that even massive sialoliths predominantly consist of hydroxyapatite. Moreover, detailed investigations indicate that levels of phytate and magnesium are decreased in patients with sialoliths [11]. Phytate and magnesium are potent crystallization inhibitors of calcium, strongly related to the daily diet, especially present in plant seeds and wheat bran, respectively. In the present study, the long-term radiographic registration suggests that the time lapse for a massive sialolith formation was less than 8 years, indicating a potential correlation with the patient's routine diet. Thus, possible evidences for the study of the etiology of massive sialoliths are addressed. Further on, deeper investigations on the biochemical formation of sialoliths are not often performed in the daily dental practice due to the high costs and limitations in accessing necessary facilities. However, biochemical investigations are relevant to the search of potential internal and external factors associated with the sialoliths formation, such as the hormonal influence. Specifically in the present case, no especial diet or systemic alterations were reported by the patient. An additional advantage of knowing the composition of a sialolith concerns the possibility of an individualized treatment, advising the patient to avoid certain dietary components, enabling an optimal postoperative follow-up.

In the present study, as consequences of the sialolithiasis, local pain, lymphadenopathy, edema, and purulent discharge were detected. Based on these findings, the patient was medicated with antibiotics during a week. Similarly, Overton et al. [12], 2012, and Combes et al. [13], 2009, treated their patients with antibiotic therapy in the immediate postoperative week. Specifically in the preset report, considering the presence of purulent discharge and the patient's allergy to Penicillin, Azithromycin 500 mg was prescribed for antibiotic coverage in combination with anti-inflammatory (Ibuprofen 600 mg) and analgesic (Paracetamol 750 mg) drugs.

Differently from the case reports already described in the medical literature, the present study is highlighted by the registration of the entire sialolith formation once no sign of ectopic mineralization was detected within the radiographs obtained out of the patient's dental records. These radiographs support the need for correctly recording dental interventions and updating patient's files, as well as early and accurately diagnosing sialolithiasis, for an optimal treatment outcome.
